# Supplementary material for: Mesothelin expression correlates with elevated inhibitory immune activity in patients with colorectal cancer
Source: Cancer Gene Ther. 2024 Aug 22;31(10):1547–58. doi: 10.1038/s41417-024-00816-1 (PMC11489080; doi:10.1038/s41417-024-00816-1)
Supplement: Supplementary file 1 — Supplemental figures [file 41417_2024_816_MOESM1_ESM.pdf]

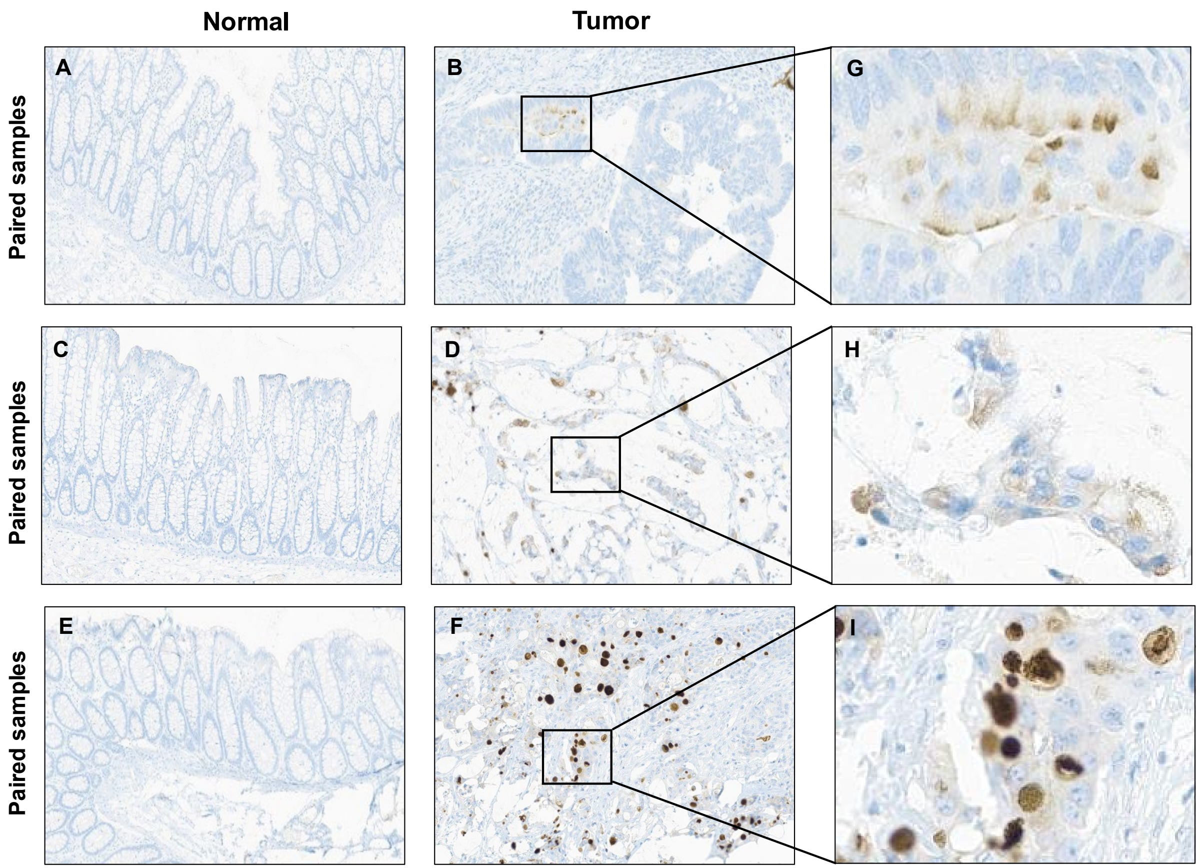

<u>Supplemental Figure 1 – Immunohistochemistry (IHC) staining images of normal paired and colorectal cancer (CRC) tumor tissue.</u> IHC analysis of matched/paired normal (A), (C) and (E) and CRC tumor tissue (B), (D), (F), respectively, was performed using 1:100 dilution of mesothelin monoclonal antibody (MN-1).

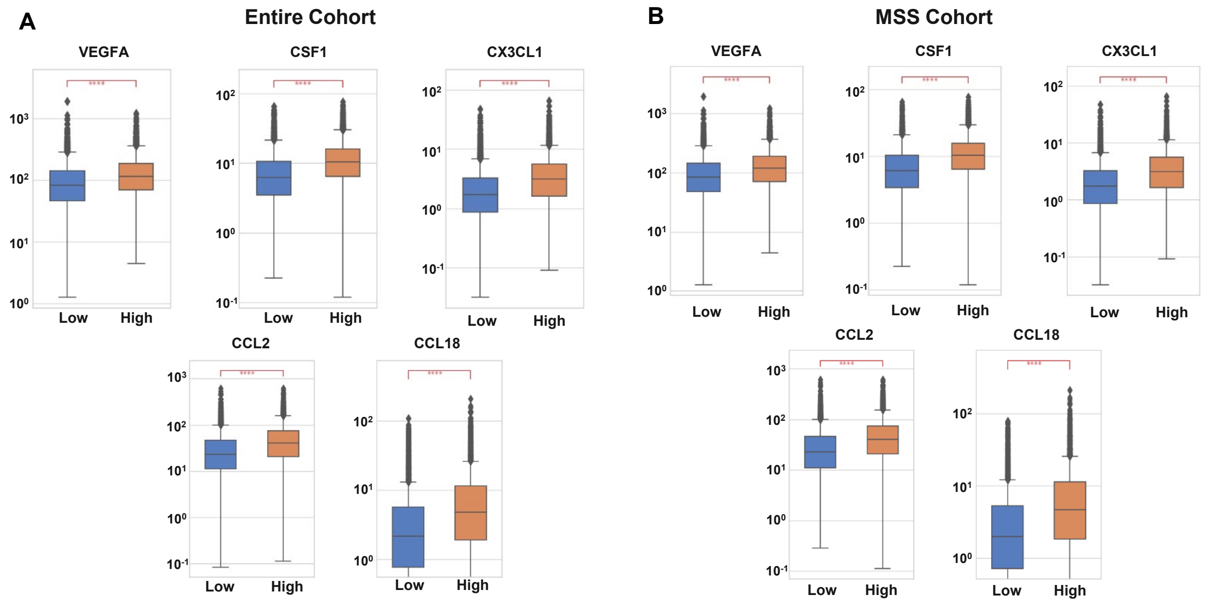

<u>Supplemental Figure 2 – Macrophage infiltration-associated cytokine and growth factor expression is increased in MSLN high CRC patients, regardless of MSI status.</u> *VEGFA, CSF1, CX3CL1, CCL2,* and *CCL18* expression were quantified via WTS in the entire cohort (A) and MSS cohort (B) for both MSLN low and MSLN high patients. Data was adjusted for multiple comparisons, where **** $q < 0.0001$.

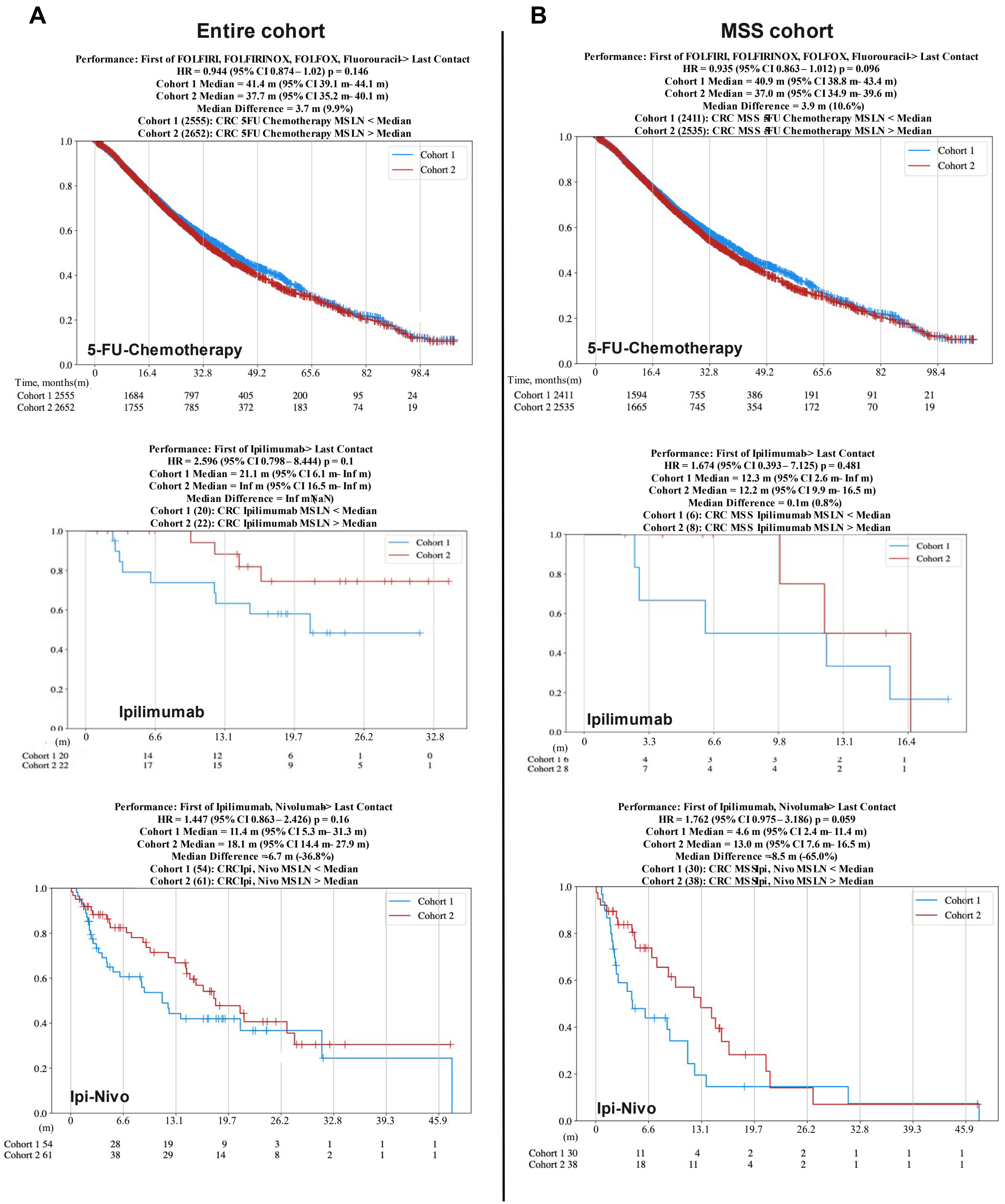

<u>Supplemental Figure 3 – Patient survival outcomes improve for *MSLN* high CRC when treated with ipilimumab and Ipi-Nivo combination.</u> Patient insurance claims data provided to Caris Life Sciences were used to generate Kaplan-Meier curves via CODEai™ data portal. Curves depict survival from time of tissue collection or first treatment to last contact for entire cohort (A) and MSS cohort (B) of CRC patients with *MSLN* low (blue/cohort 1) compared to *MSLN* high (red/cohort 2) across several therapeutic interventions.

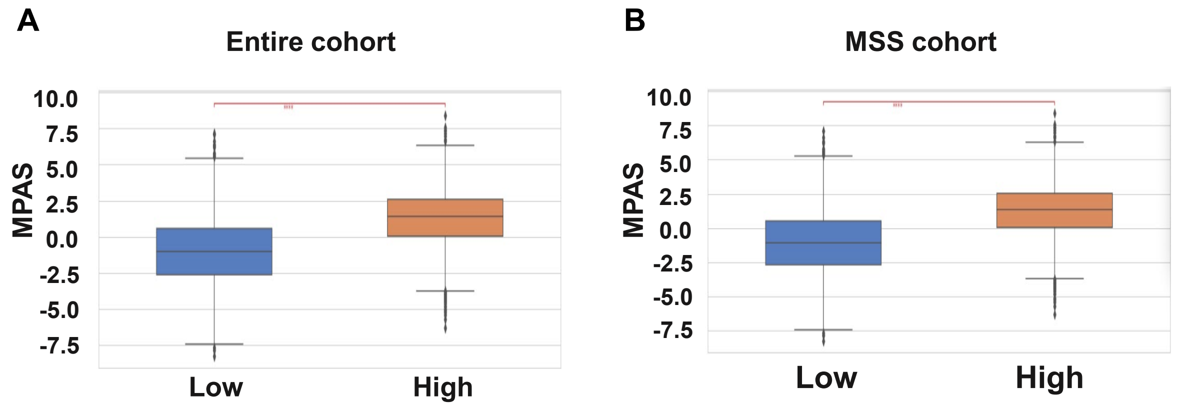

Supplemental Figure 4 – MAPK activation is significantly higher in *MSLN* high tumors, regardless of cohort. MAPK activation calculated by expression profiles of a ten-gene panel in the entire cohort (A) and MSS cohort (B) quantified using the MAPK Activation Score (MPAS). Statistical significance values were adjusted for multiple comparisons. ** $q \leq 0.01$

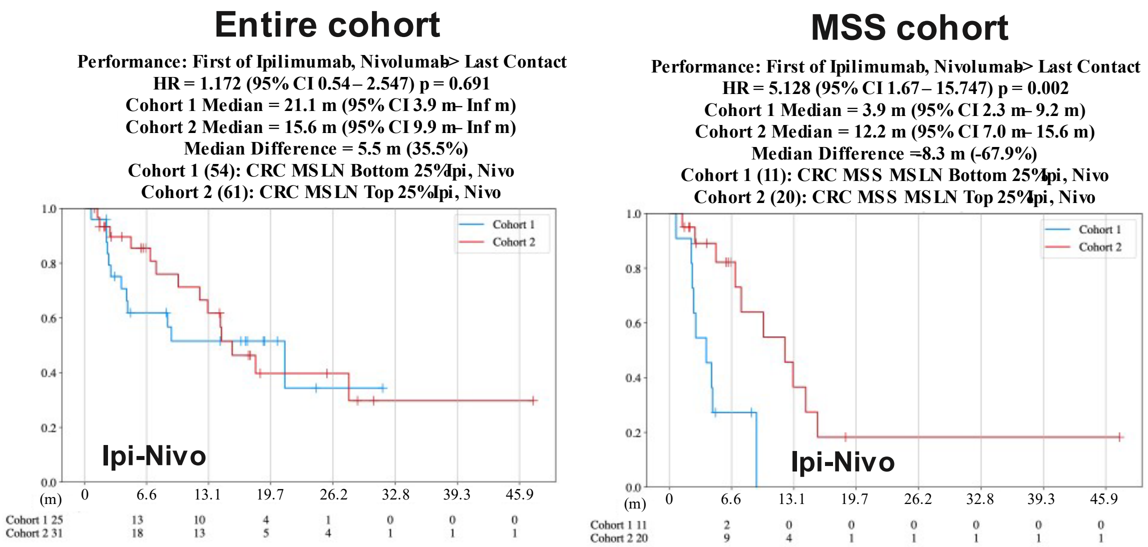

Supplemental Figure 5 – Patient survival outcomes improve in MSLN high tumors with MSS status in Ipi-Nivo combination therapy. Patient insurance claims data provided to Caris Life Sciences were used to generate Kaplan-Meier curves via CODEai™ data portal. Curves depict survival from time of tissue collection or first treatment to last contact for entire cohort (A) and MSS cohort (B) of CRC patients by the lowest 25% *MSLN* expression quartile (blue/cohort 1) and highest 25% *MSLN* expression quartile (red/cohort 2) across several therapeutic interventions.

A

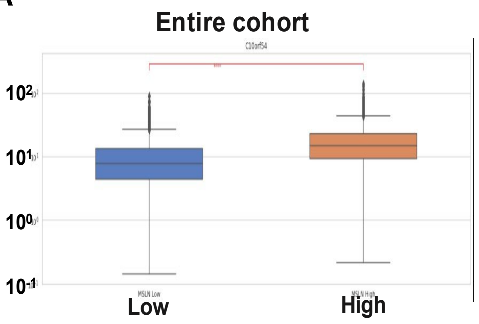

MSLN	Median	Fold change	p-value	q-value
Low	7.81	1.87	2.9e-251	2.9e-251
High	14.63			

B

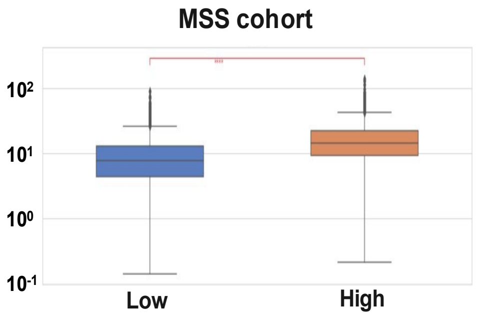

MSLN	Median	Fold change	p-value	q-value
Low	7.73	1.85	1.3e-249	1.3e-249
High	14.34			

<u>Supplemental Figure 6 – *MUC16* expression is enhanced in *MSLN* high tumors.</u> *MUC16* expression quantified as TPM via WTS analysis in the entire cohort (A) and MSS cohort (B). Statistical significance values were adjusted for multiple comparisons. ** q $\leq$ 0.01

Supplemental Table 1 – Genetic mutation prevalence quantified by NGS in *MSLN* low and *MSLN* high tumors.

Entire Cohort

Features	%MSLN Low	%MSLN High	%Change	p-value	q-value
APC	77.16	67.53	-9.63	1.70E-20	2.84E-18
KRAS	36.46	59.81	23.35	2.67E-90	1.34E-87
TP53	71.50	73.47	1.97	0.06	0.40
SMAD4	12.66	14.36	1.70	0.033	0.315
FBXW7	7.86	13.46	5.60	1.29E-14	1.61E-12
BRAF	9.22	13.12	3.90	9.32E-08	7.79E-06
PMS2	1.42	0.77	-0.65	0.021	0.238
TSC2	1.11	0.54	-0.57	0.007	0.111
MEN1	1.04	0.51	-0.53	0.009	0.128
TSC1	0.94	0.49	-0.45	0.021	0.238
MED12	0.52	1.44	0.92	0.03	0.299
U2AF1	0.03	0.24	0.21	0.011	0.154
RNF43	7.55	9.27	1.72	0.007	0.111
GNAS	1.2	6.07	4.87	2.54E-29	6.35E-27
KMT2D	6.71	5.2	-1.51	0.008	0.111
PTEN	5.82	4.41	-1.41	0.006	0.103
NRAS	3.02	4.02	1.00	0.019	0.238
SMAD2	2.24	3.9	1.66	3.93E-05	0.002
BCL9	5.22	3.59	-1.63	0.0006	0.016
CREBBP	3.87	2.29	-1.58	8.50E-05	0.004
FLCN	3.64	1.67	-1.97	1.39E-07	9.92E-06
PRDM1	0.29	0.05	-0.24	0.013	0.172
NSD2	0.44	0.06	-0.38	0.002	0.037
MAP2K1	0.59	1.24	0.65	0.003	0.06
BMPR1A	0.48	1.09	0.61	0.003	0.062
EP300	2.56	1.06	-1.50	1.42E-06	7.91E-05
STK11	0.27	0.81	0.54	0.001	0.034
MAP2K4	1.69	0.87	-0.82	0.004	0.065
BCOR	2.94	2.31	-0.63	0.09	0.51
SMARCA4	1.87	1.55	-0.31	0.30	0.87

MSS Cohort

Features	%MSLN Low	%MSLN High	%Change	p-value	q-value
APC	80.92	69.97	-10.94	8.35E-26	1.35E-23
KRAS	37.98	62.21	24.23	2.58E-89	1.25E-86
TP53	77.20	74.08	-3.12	0.003	0.062
SMAD4	13.07	14.77	1.69	0.044	0.422
FBXW7	6.22	11.90	5.68	8.38E-16	8.12E-14
BRAF	5.08	10.53	5.45	5.16E-17	6.24E-15
PMS2	0.44	0.12	-0.32	0.030	0.307
TSC2	0.84	0.26	-0.58	0.001	0.035
MEN1	0.00	0.09	0.09	0.250	0.847
TSC1	0.47	0.32	-0.15	0.314	0.915
MED12	0.40	0.69	0.29	0.529	0.940
U2AF1	0.03	0.26	0.23	0.021	0.280
RNF43	1.95	4.66	2.71	4.06E-10	2.81E-08
GNAS	0.83	5.62	4.79	4.36E-29	1.06E-26
PTEN	4.02	3.53	-0.49	0.290	0.915
NRAS	3.27	4.26	0.98	0.033	0.322
SMAD2	2.02	3.92	1.90	4.70E-06	0.0002
BCL9	1.86	1.77	-0.09	0.771	1.000
CREBBP	1.25	0.88	-0.38	0.129	0.665
FLCN	1.36	0.26	-1.10	3.77E-07	2.03E-05
PRDM1	0.12	0.00	-0.12	0.060	0.529
NSD2	0.43	0.03	-0.39	0.001	0.032
MAP2K1	0.59	1.25	0.66	0.005	0.086
BMPR1A	0.21	0.90	0.70	0.000	0.004
EP300	1.07	0.50	-0.57	0.007	0.126
STK11	0.03	0.67	0.64	8.61E-06	0.0003474
MAP2K4	1.49	0.64	-0.85	0.002	0.042
BCOR	0.66	1.42	0.76	0.002	0.051
SMARCA4	0.44	1.07	0.63	0.003	0.060

Supplemental Table 2 – Immune cell infiltration values calculated via RNA deconvolution in *MSLN* low and *MSLN* high tumors.

Entire Cohort

	MSLN	Median%	%Positive	p-value	q-value
B cell	Low	3.31	100.00	4.6E-05	9.2E-05
	High	3.44	100.00		
Mφ M1	Low	4.10	98.99	1.2E-50	1.2E-49
	High	5.06	99.79		
Mφ M2	Low	2.48	95.62	1.4E-22	7.0E-22
	High	2.87	96.55		
Monocytes	Low	0.00	0.49	0.019	0.023
	High	0.00	0.18		
Neutrophils	Low	5.69	98.03	4.3E-10	1.0E-09
	High	6.19	98.03		
NK cells	Low	3.41	99.95	0.010	0.015
	High	3.48	99.97		
T cells CD4	Low	0.00	37.57	0.621	0.621
	High	0.00	37.76		
T cells CD8	Low	0.00	40.29	0.011	0.015
	High	0.00	38.30		
Tregs	Low	1.60	93.39	0.022	0.024
	High	1.56	93.78		
DC	Low	0.49	67.85	4.3E-17	1.4E-16
	High	0.24	58.58		

MSS Cohort

	MSLN	Median%	%Positive	p-value	q-value
B cell	Low	3.36	100.00	0.01	0.02
	High	3.45	100.00		
Mφ M1	Low	3.95	98.91	1.6E-54	1.6E-53
	High	4.92	99.78		
Mφ M2	Low	2.51	96.55	1.8E-19	9.4E-19
	High	2.90	97.09		
Monocytes	Low	0.00	0.40	0.02	0.03
	High	0.00	0.11		
Neutrophils	Low	5.83	98.30	7.1E-08	1.7E-07
	High	6.27	98.15		
NK cells	Low	3.43	99.94	0.03	0.04
	High	3.48	99.97		
T cells CD4	Low	0.00	38.94	0.96	0.96
	High	0.00	38.59		
T cells CD8	Low	0.00	37.50	0.22	0.24
	High	0.00	36.72		
Tregs	Low	1.54	92.99	0.05	0.06
	High	1.51	93.43		
DC	Low	0.53	70.11	3.2E-19	1.0E-18
	High	0.26	59.56		
